# Coronavirus: Older Persons With Cancer in Italy in the COVID-19 Pandemic

**DOI:** 10.3389/fonc.2020.00648

**Published:** 2020-04-30

**Authors:** Lucia Fratino, Giuseppe Procopio, Massimo Di Maio, Saverio Cinieri, Silvana Leo, Giordano Beretta

**Affiliations:** ^1^Medical Oncology Unit, National Cancer Institute, Centro di Riferimento Oncologico, IRCCS, Aviano, Italy; ^2^Medical Oncology Department, Fondazione IRCCS Istituto Nazionale dei Tumori, Milan, Italy; ^3^Department of Oncology, University of Turin, at Ordine Mauriziano Hospital, Turin, Italy; ^4^Medical Oncology Unit, Antonio Perrino Hospital, Brindisi, Italy; ^5^Medical Oncology Unit, Vito Fazzi Hospital, Lecce, Italy; ^6^Medical Oncology Unit, Humanitas Gavazzeni, Bergamo, Italy

**Keywords:** elderly, cancer patients, COVID-19, pandemic, Italy

## Abstract

Italy is the European country that was hit first and hardest by the COVID-19 epidemic. Since February 2020, the outbreak of the epidemic disease in Italy, with fatal outcomes in up to 10% of cases, made it urgent to implement extraordinary measures to avoid a breakdown of the universal Italian national health system. The update for April 1, 2020, in Italy recorded 102,669 confirmed COVID-19 cases, with a median patient age of 63 years. The deceased patients were older people (median age 80 years) and often had a cancer diagnosis (about 20%). Thus, in the extraordinary epidemiological scenario of the COVID-19 pandemic in Italy, older persons in cancer treatment are at particularly high risk of being severely affected by COVID-19. These people face a health- and economics-related emergency that also carries cultural and ethical implications. In accordance with the measures adopted by the Italian government to limit viral transmission, several associations of Italian oncologists have taken action to update Elderly Cancer Care programs. In view of the newly emerging needs, we herein outline practical suggestions aimed at guaranteeing the best continuity to elderly cancer patients.

## Introduction

As of April 1, 2020, Italy is the country that is most affected by the COVID-19 pandemic in Europe. Within 1 month of the first case being reported in Lombardy, a highly populated and industrialized region in northern Italy, more than 100,000 infected persons and more than 12,000 deaths were documented. These unexpectedly high fatal outcomes, particularly in northern Italy, have necessitated that the scientific community addressing the COVID-19 epidemic provide, develop, and adopt extraordinary measures in order to limit viral transmission.

According to data from China—a population with heterogeneous characteristics that substantially differs from the European ones with regard to age distribution and oncological profiles—two of the main determinants of the death risk of COVID-19-infected patients are noteworthy: (1) age: the lethality steadily increases with age, with rates reaching 8% in patients between 70 and 79 years and 15% in those in their eighties; (2) the presence of comorbidities: lethality rises to 10.5% in patients with cardiovascular disease, 7.3% in diabetics, 6.3% in subjects with chronic respiratory diseases, 6% in hypertensive patients, and, finally, 5.6% in cancer patients (1, 2).

In a descriptive study of the clinical-epidemiological characteristics of 2007 Chinese patients with COVID-19, Liang et al. (3) found that cancer patients were frequently recorded among COVID-19-infected patients, that they had a more than 3-fold higher frequency of poor outcomes, and that old age was one of the major determinants of severe events (3).

## The Italian Scenario and the Characteristics of COVID-19-Positive Patients in Italy

**Figure 1:**
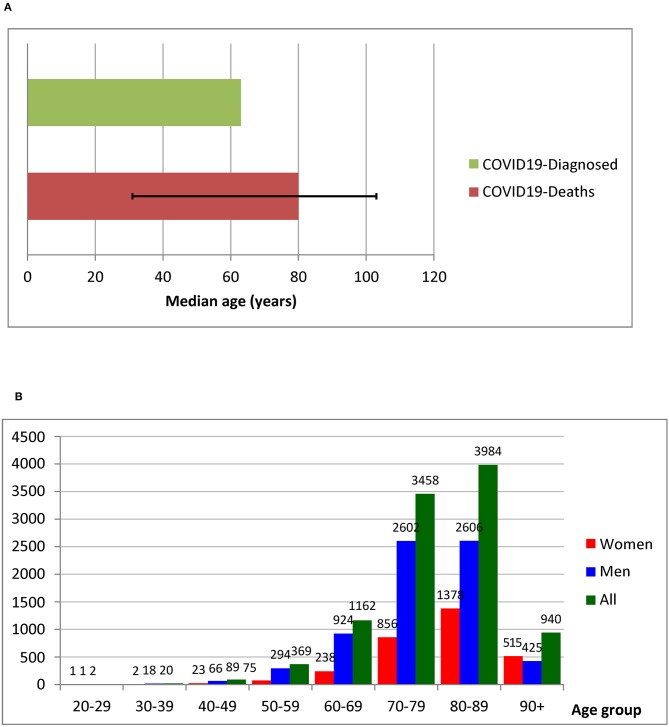
**(A)** Median age of patients with COVID-19 infection and COVID-19-positive deceased patients. **(B)** Absolute number of deaths by age group. Modified from: Characteristics of COVID-19 patients dying in Italy. Report based on available data on March 30, 2020. Istituto Superiore di Sanità, Italy.

The projected exponential growth of the COVID-19 pandemic in Italy gives rise to grave concern regarding the possibility of the universal Italian national health system not being able to cope with massive health needs in a restricted time period (4). According to the Italian National Institute of Health (Istituto Superiore di Sanità, ISS), 102,669 individuals had tested positive as of April 1, of whom 11.5% (i.e., 11,857 individuals) had already died. Based on data updated to March 30, 2020, the ISS described the demographics and clinical characteristics of 10,026 deceased patients in Italy who were positive for COVID-19. The geographical distribution of deaths showed Lombardy as the leading region in death since the beginning of the epidemic. The average age of the deceased COVID-19-positive patients is 79.4 years (median 80.0, Inter Quartile Range—IQR 75.0–85.8); 3,088 were women (30.8%). As regards the number of deaths by age group, the median age of COVID-19-positive deceased patients was ~15 years higher than the median of all patients who contracted the infection (median age of patients who died: 80 years; infected patients: 65 years) (Figure 1A). Women who died after contracting COVID-19 infection were older than men (median ages: women 84, men 79), and lethality markedly increased after the age of 70.8 years (Figure 1B). Information with regard to pre-existing conditions was also available among a subgroup of 909 deceased patients. The average number of observed comorbidities in this population was 2.7, with 51% having three or more pre-existing conditions (Table 1). Cardiovascular diseases, diabetes, and cancer were among the most frequently described conditions already present in deceased patients at the time of COVID-19 infection (5). A multivariate analysis to attribute the right weights to the effects of chronic diseases and advanced age is not yet available. In this regard, the role of well-known risk factors (e.g., smoking) for the occurrence of chronic conditions should also be considered. In addition, significant data on cancer characteristics, including type of cancer, stage, and ongoing treatment, of deceased COVID-19 patients are lacking. The Italian Association of Medical Oncology (AIOM) has already implemented specific national and international multicenter investigations (e.g., the AIOM-LCORONA study).

**Table 1:** Most common comorbidities observed in 909 COVID-19-positive deceased patients.

**Condition**	**N**.	**%**
Ischemic heart disease	249	27.4
Atrial fibrillation	209	23.0
Heart failure	149	16.4
Stroke	109	12.0
Hypertension	688	73.5
Diabetes	286	31.5
Dementia	146	16.1
Chronic obstructive pulmonary disease	166	18.3
Active cancer within the last 5 years	150	16.5
Chronic liver disease	42	4.6
Chronic renal failure	216	23.8
**Number of comorbidities**		
0	19	2.1
1	197	21.6
2	223	24.5
3 or more	470	51.7

## Practical Suggestions and Special Recommendations

**Figure 2:**
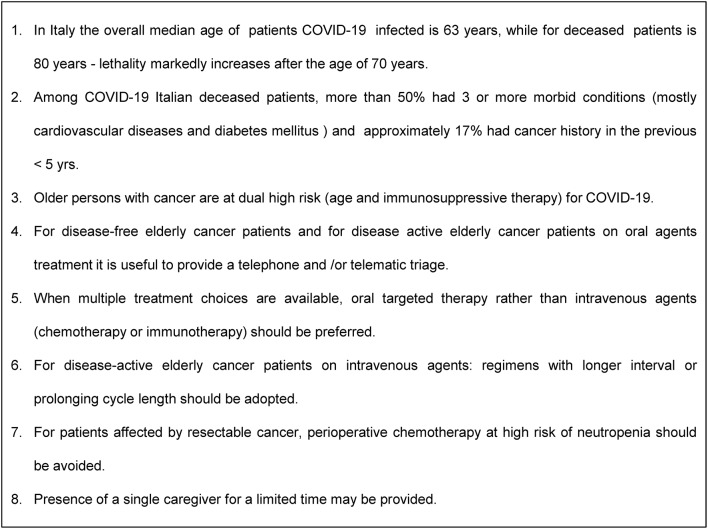
Evidence and suggestions.

The Italian demographic and epidemiological scenario is characterized by one of the highest aging indices in the world, with well-established corresponding public health programs for the geriatric population and associated chronic diseases. Accordingly, in the last few decades, the Italian medical-scientific community has developed a strong sensibility for and attention to elderly people and geriatric medicine. In the oncology field, the elderly and aging-related problems have been a clear focus of the Italian Group for Geriatric Oncology (GIOGER) since the late'90s (6, 7). Since then, Italian oncologists have been promoting clinical research focused on cancer in the elderly and also a strenuous promotion campaign against any form of ageism. Similarly, the Italian Association of Medical Oncologists (AIOM) annually publishes recommendations and guidelines for the management of elderly cancer patients (8). As a consequence, in the extraordinary emerging scenario where older persons with cancer are at dual risk (age and immunosuppressive therapy) and are the group most vulnerable to being severely affected by the COVID-19 pandemic, we face an emergency that involves not only healthcare and economics but also cultural and ethical aspects. In fact, from the organizational health perspective, the Italian government adopted extraordinary measures to limit viral transmission and to allocate resources to medical and intensive care units. From their side, Italian oncologists have taken rapid actions to update their care programs in view of the new emerging requirements. In consideration of the need to reduce the risk of SARS-CoV-2 pneumonia in elderly cancer patients, to guarantee the best continuity of care, and to contain the transmission of infected cases among patients, practical suggestions have been envisaged. They include promoting telemedicine, limiting turnover of patients, relatives, and caregivers, and providing tools and novel therapeutic strategies. Given that the continuity and timeliness of antineoplastic treatments must be guaranteed, even greater attention must be paid to the assessments that are already routinely performed in oncology, considering case by case the assessment of treatment options based on the biological characteristics of the tumor, the clinical picture, and the novel potential health risks from COVID-19 infection (9). The recommended measures are essentially aimed to: (1) favor as much as possible the social distancing of elderly cancer patients, and limit their attendance and stationing in hospitals where the risk of infection is high; (2) reduce immunodeficiency and iatrogenic treatment-related events that increase the risk of serious infection and require the consumption of medical resources. For disease-free elderly cancer patients, it is considered appropriate to postpone the follow-up (e.g., at 6–12 months), providing telephone and/or telematic triage. For disease-active patients on oral agent-treatment, it is advisable to assess clinical needs and evaluate laboratory tests by telematic triage. Moreover, according to the Italian Drug Agency (AIFA), it is now possible to extend the drug refill time. For patients with active disease receiving intravenous agents, regimens with a longer interval (e.g., 4 weeks) should be employed, while weekly regimens should be avoided when possible; in a curative and adjuvant setting, it is advisable to consider prophylactic use of growth factors for agents at high risk of myelosuppression, minimizing, if possible, the number of cycles of chemotherapy or the prolonging cycle length; when multiple treatment choices are available, oral targeted therapy rather than intravenous agents (chemotherapy or checkpoint inhibitors) may be attractive, as it requires less healthcare interaction and medical resources; for patients affected by resectable cancer, perioperative chemotherapy with high risks of neutropenia should be avoided. Regarding caregivers, in order to avoid overcrowding in waiting rooms, in the chemotherapy area, and in parking areas and in order to guarantee safe social distancing, accompanying persons are not allowed except in situations expressly authorized. For inpatients, the presence of a single companion may be provided and for a limited time (Figure 2).

## Data Availability Statement

Publicly available datasets were analyzed in this study. This data can be found here: https://www.epicentro.iss.it/coronavirus/sars-cov-2-sorveglianza-dati.

## Author Contributions

LF, GP, MD, SC, SL, and GB drafted the manuscript, analyzed data, drafted the recommendations, revised, and approved the final version.

## Conflict of Interest

The authors declare that the research was conducted in the absence of any commercial or financial relationships that could be construed as a potential conflict of interest.
